# Changes in Gender Representation in Academic Grand Rounds Speakers During the COVID-19 Pandemic

**DOI:** 10.1089/whr.2024.0174

**Published:** 2025-03-27

**Authors:** Abigail Dereje, Rahel Ghebre, Shanaz Sultan, Ben Langworthy, Michelle N. Rheault

**Affiliations:** ^1^ Center for Women in Medicine and Science, University of Minnesota Medical School, Minneapolis, Minnesota, USA.; ^2^ Department of Obstetrics and Gynecology, University of Minnesota Medical School, Minneapolis, Minnesota, USA.; ^3^ Department of Medicine, University of Minnesota Medical School, Minneapolis, Minnesota, USA.; ^4^ Division of Biostatistics, School of Public Health, University of Minnesota, Minneapolis, Minnesota, USA.; ^5^ Department of Pediatrics, University of Minnesota Medical School, Minneapolis, Minnesota, USA.

**Keywords:** academic medicine, women in medicine, gender equity, academic promotion

## Abstract

**Introduction:**

Women are historically underrepresented as grand rounds speakers in US medical schools regardless of department. We hypothesized that the representation of women as grand rounds speakers would increase after the widespread adoption of virtual grand rounds during the COVID-19 pandemic.

**Methods:**

We reviewed publicly available or individually provided grand round speaker lists for 2019, 2021, and 2022 from representative medical departments, surgical departments, and basic science departments of our local institution and the top 10 academic medical schools based on their national ranking according to the 2022 Blue Ridge Institute for Medical Research Rankings of NIH Funding. Speaker gender was determined based on name and publicly available biographies.

**Results:**

In total, we identified 1995 speaking engagements from 10 institutions. All but six talks delivered post-COVID-19 pandemic were in hybrid or virtual-only format compared to exclusively in-person sessions pre-COVID-19 pandemic. Before the COVID-19 pandemic, women accounted for 37.3% of invited speakers compared to 45.5% post-COVID. This increase in the representation of women as grand rounds speakers was consistent across all academic ranks and most departments.

**Conclusions:**

We propose that departments, particularly those with fewer women at baseline, should continue to offer at least some virtual grand rounds opportunities throughout the year with a goal of increasing the diversity of speakers and improving the access of their faculty to women speakers.

## Introduction

Women in academic medicine continue to lag behind their male peers in rates of advancement to senior positions. Despite demonstrated progress in the number of women entering medical school, women continue to be underrepresented in rates of promotion to full professor and other senior leadership roles. A report by the Association of American Medical Colleges found that women made up 49% of assistant professors, 43% of associate professors, and only 29% of full professors and 27% of Deans in 2023–2024.^1^ Among the many factors that influence the promotion of faculty in academic medicine, externally invited speaking opportunities serve as a metric of national and international faculty reputation and validate the merit of their scholarship as measured by peers.

Grand rounds (GR) speakers are invited for an array of reasons, including subject matter expertise or session themes. An invitation to speak at GR at another academic institution is generally regarded as an honor and acknowledges the speaker’s prominence in their field. It can provide an opportunity to disseminate one’s research and may enhance one’s national reputation. Seniority and a faculty member’s network can play an important role in being selected as an invited speaker. This may place women at a disadvantage in obtaining speaking invitations due to the generational gender disparity in the medical field. Historically, women have been underrepresented as speakers in GR and other department seminar series. A 2017 US study evaluating gender representation in GR speakers among nine clinical specialties found a consistent underrepresentation of women; women accounted for only 28.3% of all speakers despite accounting for 41% of medical school faculty overall.^2^ A more recent article demonstrated similar findings from Mayo Clinic’s Minnesota, Florida, and Arizona campuses with women accounting for only 22.7% of speakers.^3^ Additionally, further analysis based on specialty found the representation of women GR speakers was lower than men in 14 of the 18 departments reviewed. These findings remain consistent not only within the United States but also across other countries. A report from Canada found that on average, women were represented as GR speakers 17% less often as compared to men.^4^ These studies demonstrate the widespread and consistent disparity in the representation of women as speakers in academic medicine.

The emergence of the COVID-19 pandemic caused unprecedented change in the medical field, including a transition from in-person to virtual GR. There have been advantages to the switch to this format. A single-center study found that virtual GR attracted a larger audience compared to in-person.^5^ Other studies have confirmed these findings and also demonstrate improvement in the geographic diversity of chosen speakers.^6,7^ Studies of conference attendance in STEM fields outside of medicine demonstrated the representation of women at virtual conferences increased by 253% after the transition to virtual conferences compared to 121% for men at the same events.^8^ Women may be more likely to accept virtual speaking opportunities given the challenges of travel, especially if they have caregiving obligations, thus the change to virtual GR at many centers may benefit women.

In this study, we sought to determine the impact of the transition from in-person to virtual GR during the COVID-19 pandemic. We hypothesized that the representation of women as speakers at medical school GR presentations would increase.

## Methods

### Study design and population

A retrospective cross-sectional study of invited speakers (internal and external) was conducted from a diverse geographical selection of medical schools and departments across the United States.

This study population included the top 10 academic medical schools based on their national ranking according to the 2022 Blue Ridge Institute for Medical Research Rankings of NIH Funding (https://brimr.org/, accessed June 13, 2023) which included the following: University of California San Francisco, University of California Los Angeles, University of California San Diego, Vanderbilt University, Columbia University, Duke University, Johns Hopkins University, University of Pittsburgh, University of Pennsylvania, and Stanford University. We also included our home institution the University of Minnesota to inform local policy. We chose to collect data from representative medical departments (pediatrics, internal medicine, and neurology), surgical departments (obstetrics/gynecology, urology, and orthopedics), and basic science departments (microbiology, neuroscience, and genetics). This study did not require formal IRB approval as the study met IRB exemption based on the University of Minnesota IRB guidelines. Data collection was conducted from July 2023 to August 2023.

The study collected data on invited speakership opportunities defined as GR, seminars, or specially designated lectures that have regularly scheduled presentations throughout the academic calendar year. The archived list of individual speakers was obtained from all medical school departments that hosted GR and other speakership opportunities in calendar years 2019 (defined as “pre-COVID”), 2021, and 2022 (“post-COVID”) either *via* department websites or direct email request. One follow-up email was sent to each department contact if no response was received. In 2020, during the height of the COVID-19 global pandemic, many universities and organizations significantly cut down on their educational activities, thus 2020 data were excluded from this study.

Faculty information including gender and academic rank for each speaker, as disclosed on their publicly available biographical profile, was collected. Gender was designated as woman, man, or unknown based on pronouns, name, and appearance in biographical photos. The hosting medical school, department, and month and year of each presentation were noted. Data entry was entered into Excel and cross-checked with the original communication for accuracy.

In order to compare representation between the pre-COVID and post-COVID periods, we calculated the proportion of women speakers, using the aforementioned criteria, in the pre-COVID and post-COVID years. In addition to comparing the proportion of women across all collected data, we also calculated the proportion of women GR speakers’ pre-COVID and post-COVID split out by academic rank and department. Finally, we compared the proportions of women grand round speakers by year.

To test for differences between pre-COVID and post-COVID, we used a mixed-effects logistic regression model with the gender of the speaker as the outcome and a random effect for each department within each school. We also include a fixed effect for whether the speaker was during the pre-COVID or post-COVID time period. The exponentiated coefficient for this variable is interpreted as the multiplicative increase in the odds of a GR speaker from a given department being a woman.

## Results

Among the 11 academic medical schools, a total of 28 departments had publicly available GR speaker lists. An additional 54 departments across 11 academic institutions were invited to share their invited speakership rosters *via* email. Six (11%) of these departments provided data for a total of 34 departments included in this analysis. No data were available or received from the University of Pennsylvania. In total, we identified 1995 speaking engagements from 10 institutions, with 474 performed pre-COVID-19 pandemic (January 2019–December 2019) and 1521 talks delivered post-COVID-19 (January 2021–December 2022). All but six talks delivered post-COVID-19 pandemic were in hybrid or virtual-only format contrary to exclusively in-person sessions pre-COVID-19 pandemic. Before the COVID-19 pandemic, women accounted for 37.3% of invited speakers compared to 45.5% post-COVID (Table 1). The odds ratio for female speakers was 42% higher in the post-COVID-19 period (*p* = 0.003).

**Table 1.** Grand Rounds Speakers Demographics

	Pre-COVID-19 pandemic (2019), *n* = 474	Post-COVID-19 pandemic (2021–22), *n* = 1521
Speaker gender		
Woman	177 (37.3%)	692 (45.5%)
Man	296 (62.4%)	828 (54.4%)
Unknown/missing	1 (0.2%)	1 (0.1%)
Academic rank		
Assistant professor	87 (18.4%)	340 (22.4%)
Associate professor	109 (23%)	267 (17.6%)
Professor	217 (45.8%)	739 (48.6%)
Other	1 (0.2%)	2 (0.2%)
Unknown/Missing	60 (12.7%)	173 (11.4%)
Format		
In-person	474 (100%)	6 (0.4%)
Hybrid	0 (0%)	208 (13.7%)
Virtual	0 (0%)	1307 (85.9%)

**FIG. 1.**
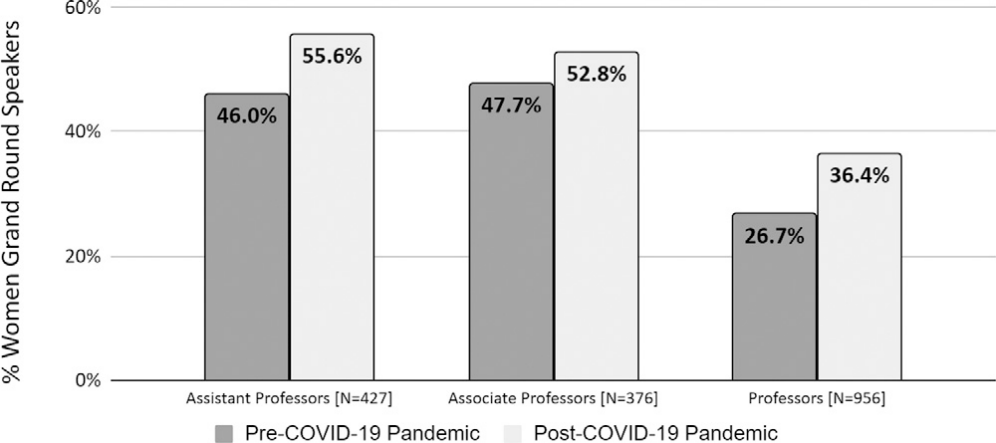
Percentage of women grand round speakers by academic rank. The proportion of women giving grand rounds presentations increased at all academic ranks after the COVID-19 pandemic.

An analysis of the distribution of speaking engagement based on faculty academic rank noted that the proportion of talks given by women increased at all ranks in the post-COVID era (Fig. 1). GR delivered by women Assistant Professors increased from 46.0% pre- to 55.6% post-COVID (odds ratio [OR] = 1.44, *p* = 0.20). The proportion of women GR speakers at the Associate Professor level increased from 47.7% to 52.8% (OR = 1.15, *p* = 0.57), while the proportion of women GR speakers at the Professor level increased from 26.7% to 36.4% (OR = 1.57, *p* = 0.02).

**FIG. 2.**
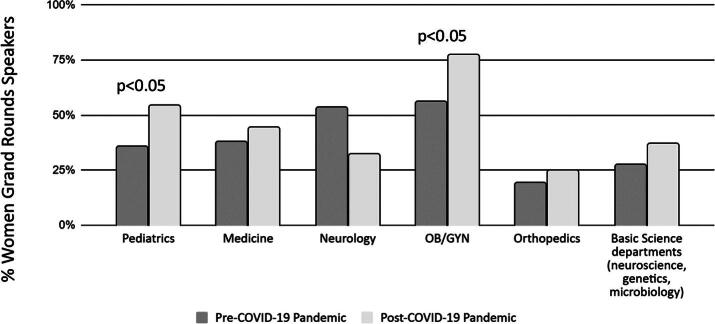
Percentage of women grand rounds speakers by department. Pediatric and OB/GYN departments demonstrated a significant increase in women grand round speakers after the COVID-19 pandemic. Medicine, orthopedics, and basic science departments also demonstrated increases; however, these were not statistically significant.

We next evaluated the differences by the department (Fig. 2, Table 2). Data from only a single urology department were available, thus these data were not analyzed separately. Pediatric and obstetrics and-gynecology (OB/GYN) departments showed significant increases in the proportion of women speakers from pre to post-COVID-19. Pediatric departments showed an increase of 18.8% in the proportion of women GR speakers from the pre to post-COVID19 era (OR = 2.18, *p* = 0.003), while OB/GYN departments showed a 21.1% increase (OR = 2.42, *p* = 0.01). Post-COVID-19 pandemic, women accounted for 54.5% of Pediatric GR speakers which remains below the overall proportion of women who make up pediatric faculty (62%).^1^ OB/GYN departments in contrast showed an increase in representation of women GR speakers that exceeded the overall faculty representation of 69% women.^1^ The proportion of women GR speakers in Departments of Medicine (OR = 1.29, *p* = 0.19) and Orthopedics (OR = 1.24, *p* = 0.69) both increased by between 5% and 7%. The proportion of women GR speakers in Neurology departments actually decreased by over 20% (OR = 0.73, *p* = 0.42), although this difference is not statistically significant, potentially in part due to the smaller sample size for this subgroup. Basic science departments (OR = 1.44, *p* = 0.20) also showed a nearly 10% increase in the percentage of women GR speakers although these did not reach statistical significance.

**Table 2.** Proportion of Women Speakers Pre- and Post-COVID-19 Pandemic by Department Versus Overall Representation of Women in the Field

Department	Pre-COVID-19 pandemic	Post-COVID-19 pandemic	Odds ratio (*p* value)	Proportion of women faculty^*^
Pediatrics (*n* = 348)	35.7%	54.5%	2.18 (0.003)	62%
Medicine (*n* = 536)	37.9%	44.3%	1.29 (0.19)	43%
Neurology (*n* = 267)	53.7%	32.3%	0.73 (0.42)	44%
OB/GYN (*n* = 247)	56.0%	77.2%	2.42 (0.01)	69%
Orthopedics (*n* = 221)	19.4%	24.9%	1.24 (0.69)	22%
Basic Science Departments (*n* = 309)	27.6%	37.0%	1.44 (0.20)	31–43%

^*^ Data from The State of Women in Academic Medicine 2023–2024 report from the AAMC.

AAMC, Association of American Medical College.

**FIG. 3.**
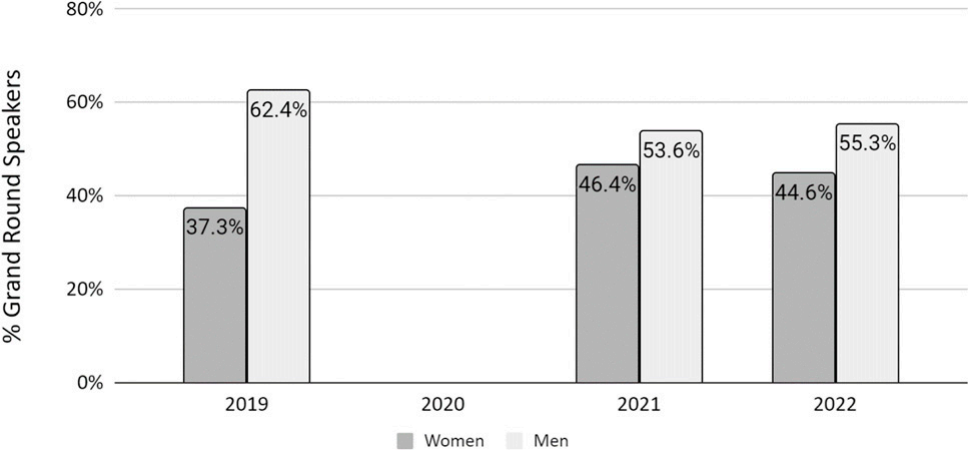
Percentage of grand rounds speakers by gender over time. The gains in the percentage of women giving grand rounds presentations after the COVID-19 pandemic were sustained over time.

Finally, we assessed the increase in the proportion of women GR speakers observed in the post-COVID era for 2021 and 2022 (Fig. 3). The percentage of women GR speakers was similar in 2021 and 2022, showing a sustained increase compared to the pre-COVID year.

## Discussion

A prior single-center study of gender representation in academic GR found that the COVID-19 pandemic did not appear to influence the gender representation of speakers.^9^ In contrast, this study demonstrates an increase in the representation of women as GR speakers across all academic levels in 2021 and 2022 compared to pre-COVID-19 pandemic year of 2019 using a geographically diverse sample. These findings suggest that the increase in virtually held GR may have allowed for more women to participate in these presentations, a benefit of transition brought about by the COVID-19 pandemic. We hypothesize that women may be more likely to accept a virtual speaking invitation compared to an in-person invitation requiring travel due to work–family conflict. Multiple studies have documented work–family conflict, defined as challenges experienced when an individual attempts to meet the demands of both work and family, as one reason for slower career progression and lower rates of promotion for women in academic medicine.^10^ Prior to the COVID-19 pandemic, women physician–scientists with children spent 8.5 more hours per week on domestic duties or childcare compared to men.^11^ During the COVID-19 pandemic, these disparities were further exacerbated, and work–family conflict was higher for women academic clinician front-line workers compared to men.^12^ These family responsibilities may limit the availability of women to travel for GR and may in part account for the increases seen in the representation of women as virtual speakers. A similar experience may exist across all genders who experience high work–family and family–work conflict.

After the COVID-19 pandemic, several specialties achieved an increase in the proportion of women GR speakers that were at or above the overall proportion of women in that specialty including medicine, OB/GYN, orthopedics, and basic science departments. While pediatrics departments showed significant gains, their representation of women GR speakers remains below the proportion of women in pediatrics departments.

This study has several limitations. The data were gathered from the top 10 medical institutions in the United States as identified by the 2022 Blue Ridge Institute for Medical Research Rankings of NIH Funding; however, these data may not sufficiently represent all US medical schools. Additionally, our access to data was restricted, as not all information was available online and not all departments agreed to share their GR speaker lists. Thus we cannot exclude bias introduced by which departments agreed to share information. We recognize that gender is a nonbinary concept and did not ask individuals to self-define their gender for this study. Determination of the speaker’s gender using names and personal biographies could potentially mis-gender individuals. Considering that the GR presentations delivered may not reflect the invitations to provide the talk, it is important to recognize that the circumstances of COVID-19 may have limited faculty from accepting speaking invitations, and this may also influence the final speaker numbers. We were unable to determine the composition of the GR chairs or committees who determine the GR agenda at each institution. Prior studies have demonstrated that the presence of women as chairs increases the likelihood of women appearing as speakers.^13^ Finally, we cannot confirm that the transition to virtual GR was the only reason that accounted for the increase in the representation of women.

The underrepresentation of women as invited speakers is a potential actionable target for providing equitable opportunity for academic advancement. We demonstrate an increase in the proportion of women as grand round speakers after the widespread introduction of virtual GR after the COVID-19 pandemic at US medical schools. We propose that departments, particularly those with fewer women at baseline, should continue to offer at least some virtual GR opportunities throughout the year with a goal of increasing the diversity of speakers and improving the access of their faculty to women speakers.

## Abbreviations Used

GR: Grand Rounds
OB/GYN: Obstetrics/gynecology
OR: Odds ratio
US: United States
